# *Listeria monocytogenes*: survival and adaptation in the gastrointestinal tract

**DOI:** 10.3389/fcimb.2014.00009

**Published:** 2014-02-05

**Authors:** Cormac G. M. Gahan, Colin Hill

**Affiliations:** ^1^Alimentary Pharmabiotic Centre, University College CorkCork, Ireland; ^2^School of Microbiology, University College CorkCork, Ireland; ^3^School of Pharmacy, University College CorkCork, Ireland

**Keywords:** *Listeria*, stress, acid, bile, gastrointestinal, virulence, pathogenesis, infection

## Abstract

The foodborne pathogen *Listeria monocytogenes* has the capacity to survive and grow in a diverse range of natural environments. The transition from a food environment to the gastrointestinal tract begins a process of adaptation that may culminate in invasive systemic disease. Here we describe recent advances in our understanding of how *L. monocytogenes* adapts to the gastrointestinal environment prior to initiating systemic infection. We will discuss mechanisms used by the pathogen to survive encounters with acidic environments (which include the glutamate decarboxylase and arginine deiminase systems), and those which enable the organism to cope with bile acids (including bile salt hydrolase) and competition with the resident microbiota. An increased understanding of how the pathogen survives in this environment is likely to inform the future design of novel prophylactic approaches that exploit specific pharmabiotics; including probiotics, prebiotics, or phages.

## Introduction

*Listeria monocytogenes* is a Gram positive foodborne pathogen capable of causing infection of the fetus in pregnant women and meningitis, meningoencephalitis or febrile gastroenteritis in non-pregnant individuals. The pathogen continues to cause large common-source outbreaks from ready-to-eat food sources and represents a significant cause of food-related mortality (Jackson et al., 2011; Mccollum et al., 2013). The pathogen exists in the environment as a saprophyte and can access the human food chain either directly or through infection or carriage in farm animals (zoonotic disease). *Listeria* possesses a number of molecular mechanisms with which to adapt to the different stages of the pathogenic lifecycle (reviewed previously in Gahan and Hill, 2005; Gray et al., 2006; Sleator et al., 2009; Camejo et al., 2011; Cossart, 2011). Here we focus upon recent advances in our understanding of how *L. monocytogenes* adapts to the environment of the gastrointestinal (GI) tract and makes the transition from saprophyte to pathogen.

## Potential for faecal or gall bladder carriage

*L. monocytogenes* faecal carriage in farm animals has been reported as 21.3% in cattle and 1.5% in sheep (Esteban et al., 2009), while it can be detected in the faeces of 2.1% of asymptomatic individuals in human population studies (Cobb et al., 1996). An interesting study followed the appearance of *L. monocytogenes* in the faeces of three human volunteers (one male, two females) over approximately 1 year (Grif et al., 2003). *L. monocytogenes* was isolated in 31 of the 868 (3.57%) stool samples analyzed, which suggests between 5 and 9 annual exposures to *L. monocytogenes* per person. In the majority of cases *L. monocytogenes* carriage was transient, lasting between 1 and 4 days (Grif et al., 2003).

Active systemic infection in mice following intravenous inoculation results in faecal shedding of *L. monocytogenes* that can last for up to 9 days post-infection (Nichterlein et al., 1994). It is significant that *L. monocytogenes* can colonize the murine gall bladder following oral or intravenous administration (Hardy et al., 2004; Bron et al., 2006). It can also be isolated from the gall bladder in infected guinea pigs (Jensen et al., 2008) and turkeys (Huff et al., 2005), but not in infected sheep (Zundel and Bernard, 2006). There is also evidence suggesting that *L. monocytogenes* may be a rare cause of human cholecystitis (infection of the gall bladder) (Allerberger et al., 1989; Descy et al., 2012; Bruminhent et al., 2013). In mice efficient growth of *L. monocytogenes* in the gall bladder results in rapid shedding into the GI tract and faeces (Hardy et al., 2006). Growth in this bile-rich environment may therefore provide a significant source of faecal shedding during infection. We have recently demonstrated that gall bladder bile actually serves as an efficient growth environment for a number of bacterial species (including non-pathogens), suggesting that *L. monocytogenes* is not unusual in its ability to grow in this environment (Dowd et al., 2011). The ability of the pathogen to cross epithelial barriers may make this a potential site for bacterial replication, a phenomenon which is perhaps further influenced by specific host conditions (Dowd et al., 2011; Bruminhent et al., 2013). Furthermore, to our knowledge the possibility of ascending bile duct infection by *L. monocytogenes* has not been investigated.

## Insights into stress adaptation in the GI tract

Given the evidence of transient faecal carriage of *L. monocytogenes* (Grif et al., 2003), it is likely that the pathogen is a common allochthonous passenger in the human gut rather than an autochthonous commensal. It is clear that the alternative sigma factor, Sigma B, plays a significant role in adaptation to the gastrointestinal environment (Sleator et al., 2009; Toledo-Arana et al., 2009). Mutation of *sigB* results in decreased virulence of *L. monocytogenes* when administered orally to guinea pigs or mice (Nadon et al., 2002; Garner et al., 2006). However, systemic infection is not affected. Transcriptomic analyses have identified the members of the Sigma B regulon, the exact components of which may differ marginally between *L. monocytogenes* isolates (Hain et al., 2008; Sleator et al., 2009), and which contain genes encoding systems for bile, acid, and salt adaptation (Hain et al., 2008; Oliver et al., 2009; Chaturongakul et al., 2011; Mujahid et al., 2013).

A comprehensive transcriptomic analysis of listerial growth both *in vivo* and *in vitro* was carried out using tiling arrays in order to map the expression of genes and small RNAs (sRNAs) during the passage from saprophytism to virulence (Toledo-Arana et al., 2009). The study identified 50 sRNAs as well as antisense RNAs that point to an added layer of regulatory complexity. The work definitively established that Sigma B is the predominant regulator of virulence genes and survival factors in the GI tract, with PrfA acting as the main regulator of virulence genes in blood (Toledo-Arana et al., 2009). Adaptation to the murine GI tract resulted in significant reshaping of the transcriptional profile, with the upregulation of 437 genes and the downregulation of 769 genes, many of which are regulated by Sigma B (including *inlA, inlB, inlH*, and *bsh*) (Toledo-Arana et al., 2009).

Further studies of *L. monocytogenes* gene expression profiles in the GI tract of germ-free mice or mice mono-colonized by *Lactobacillus* species have provided insights into how the pathogen adapts to the physical and chemical environment of the gut (Archambaud et al., 2012). In an elegant experimental setup transgenic mice optimized for listerial infection (E-cad^hum^ mice) were mono-colonized with either *Lb. paracasei* CNCM I-3689 or *Lb. casei* BL23 prior to oral infection with *L. monocytogenes*. Passage from broth to the GI tract resulted in the upregulation of 520 protein coding genes and seven non-coding sRNAs, whereas 523 genes and three sRNAs were downregulated. The presence of *Lactobacillus* spp. resulted in enhanced effects upon numerous gene systems suggesting a major influence of the microbiota upon gene expression in *L. monocytogenes*. Many of the listerial genes that were positively influenced by the microbiota are potentially involved in adaptation to the gut environment and include genes encoding pathways involved in ethanolamine and propanediol metabolism that have been implicated in the gastrointestinal survival of *Listeria* (Cummins et al., 2013) and other bacteria (Sriramulu et al., 2008; Archambaud et al., 2012).

Functional genetic approaches have been employed to determine the key systems required for survival in the GI tract (Sleator et al., 2009). We recently employed a mariner transposon-based signature-tagged mutagenesis approach to identify loci likely to be play a role during the gastrointestinal phase of *L. monocytogenes* infection (Cummins et al., 2013). The study determined the importance of a gene encoding a Sigma B-regulated internalin-like protein (equivalent to *lmo0610*) (Mcgann et al., 2008) for pathogenesis via the gastrointestinal route of infection. A mutant in an enzyme putatively involved in propanediol utilization was also unable to infect mice efficiently via the oral route, providing further evidence that ethanolamine/propanediol pathways are important for listerial growth in the GI tract (Archambaud et al., 2012). We also identified a number of putative regulators, an iron uptake system (Jin et al., 2006) and metabolic systems required for colonization of the GI tract and subsequent infection using our approach (Cummins et al., 2013).

## Adaptation to low pH

During passage through the GI tract *L. monocytogenes* encounters low pH environments in both stomach and duodenum. In humans the low pH of the stomach provides a significant barrier to *L. monocytogenes* infection and patients taking medications which reduce gastric acid (including proton pump inhibitors) are at increased risk of infection (Bavishi and Dupont, 2011). *Listeria* possesses a number of systems that regulate intracellular pH during exposure to acidic environments (reviewed in Smith et al., 2013 and Ryan et al., 2008).

**Figure 1:**
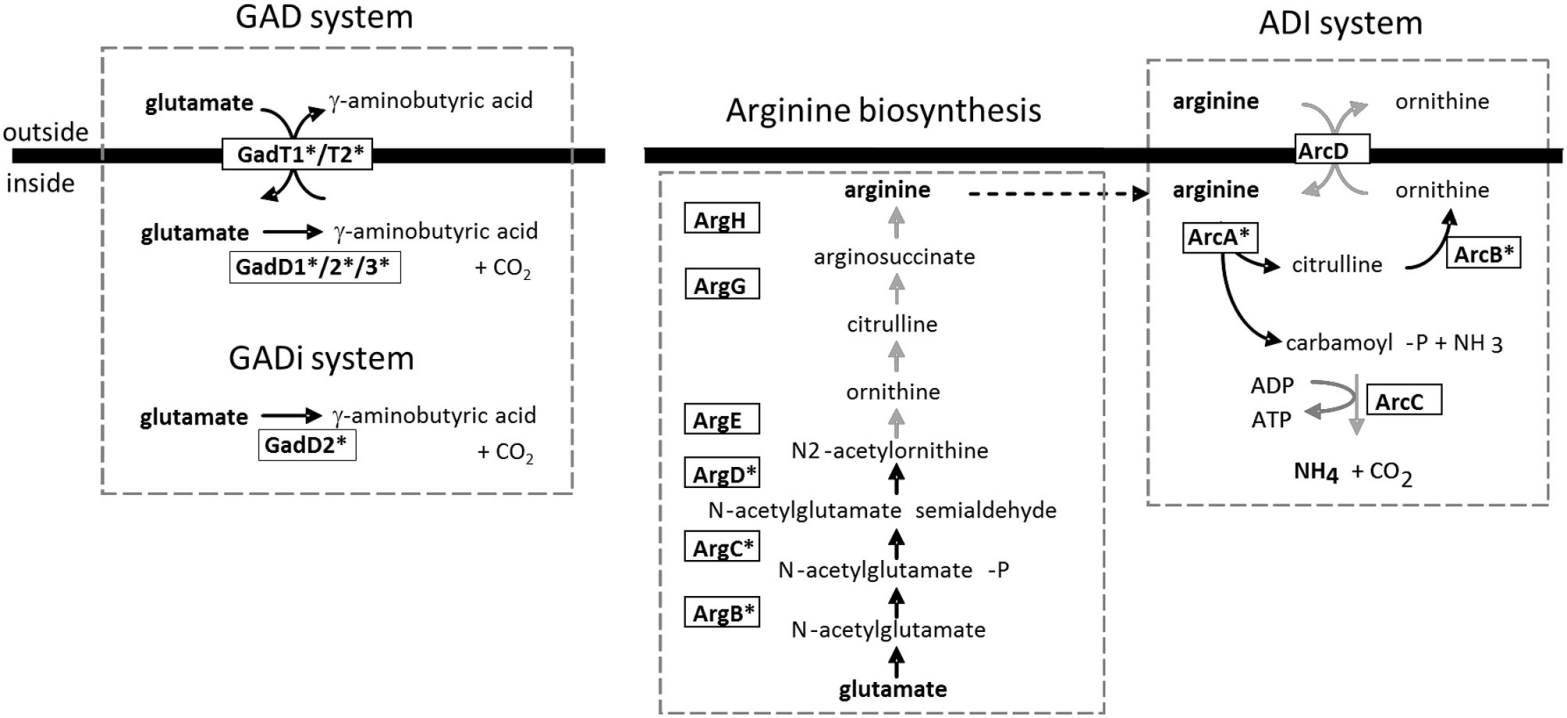
**Two major acid tolerance mechanisms in *L. monocytogenes***. The GAD system is proposed to function through the import of glutamate into the cell (via GadT1 or GadT2) which is converted to GABA via Gad enzymes (GadD1, D2, or D3) in the cytoplasm. The GABA is then thought to be exported from the cell by means of the antiport systems (GadT1/T2). The consumption of protons during the reaction is contributes to the reduction in intracellular hydrogen ion concentration (Cotter et al., 2001). Karatzas et al. (2012) have recently proposed the existence of an intracellular GAD_i_ system that operates in the absence of added glutamate. The ADI system has also been proposed to operate via utilization of intracellular arginine as well as via arginine import (Ryan et al., 2009). The arginine biosynthetic pathway is shown. Black arrows indicate genes that are upregulated during growth of *Listeria* in the GI tract of germ-free mice and further upregulated through *in situ* exposure to *Lactobacillus* spp. (Archambaud et al., 2012). In such cases the enzyme is highlighted with an asterisk.

The glutamate decarboxylase (GAD) system is particularly important for mediating pH homeostasis. The GAD system in *L. monocytogenes* is complex and comprises two glutamate/GABA antiporters (GadT1 and GadT2) and three glutamate decarboxylase enzymes (GadD1, GadD2, and GadD3) (Figure 1). A recent study demonstrated that the GadD2/T2 and GadD3 systems are present in all strains analyzed but the GadD1/T1 system is present in only 36.6% of strains analyzed and is absent from serotype 4b and lineage IV strains (Chen et al., 2012). Indeed the *gadD1* and *gadT1* genes are present on a five-gene stress survival islet (SSI-1) (Ryan et al., 2010) that also contains a gene encoding a putative penicillin V acylase that is required for maximum bile tolerance (Begley et al., 2005b; Ryan et al., 2010). Deletion of SSI-1 from *L. monocytogenes* strain LO28 reduced the acid and salt tolerance of the strain and significantly affected ability to grow in foods, suggesting the importance of SSI-1 for survival during the infectious cycle (Ryan et al., 2010).

Clearly the GAD system plays a significant role in pH homeostasis in *L. monocytogenes* as mutants in *gadT2/D2* (Cotter et al., 2001) are significantly compromised in acid tolerance. Evidence suggests that *GadD1/T1* is important for growth in mildly acidic environments rather than under extremes of acid stress (Cotter et al., 2005). The ability of individual strains to grow at mildly acidic pH correlates with the presence of the *GadD1/T1* present on SSI-1 (Cotter et al., 2005). Recently it has been demonstrated that GABA may accumulate in *L. monocytogenes* under minimal growth conditions, providing evidence for an intracellular GAD system (GAD_i_) that operates separately from the canonical extracellular GAD (GAD_e_) system (Karatzas et al., 2010, 2012). Interestingly all elements of the GAD system are transcriptionally upregulated in *Listeria* during colonization of the GI tract (Archambaud et al., 2012).

*L. monocytogenes* strains also possess an arginine deiminase (ADI) pathway and an agmantine (AgDI) deiminase system that contribute to pH homeostasis (Ryan et al., 2008; Smith et al., 2013). The ADI system is not present in the non-pathogenic species *L. innocua*. The ADI system operates through the catabolism of arginine to produce ornithine and ammonium ions (NH^+^_4_) which protect the cells from low pH (Figure 1). In a typical bacterial ADI system extracellular arginine feeds the ADI system via an arginine/ornithine antiporter (Cotter and Hill, 2003). However, we noted that the *L. monocytogenes* ADI contributes to pH homeostasis in the absence of extracellular arginine and we have evidence to suggest that the system also utilizes arginine that is synthesized via the cellular arginine biosynthetic pathway (encoded by the *arg* genes). In *L. monocytogenes* LO28 the arginine catabolic genes of the ADI system (including *arcA* and *arcC*) and the arginine biosynthetic genes (including *argG*) are induced by low pH and anaerobicity and are under the influence of the transcriptional regulator ArgR (Ryan et al., 2009). The data suggest that *L. monocytogenes* may synthesize arginine under anaerobic conditions to regulate cellular homeostasis through the ADI system (Ryan et al., 2009). It is interesting to note that the ADI system is also regulated by Sigma B (Hain et al., 2008) and the genes encoding both the arginine deiminase (*arcA*) and enzymes of the biosynthetic pathway (including *argB, argC*, and *argD*) are significantly induced in the GI tract of germ-free mice and are further induced by the presence of lactobacilli (Archambaud et al., 2012).

## Adaptation to bile acids

Bile acids are produced from cholesterol in the liver, stored in the gall bladder and are released into the duodenum postprandially (Begley et al., 2005a). Bile acids can disrupt bacterial membrane structure, cause dissociation of membrane proteins, induce DNA damage and trigger oxidative stress in bacterial cells (Begley et al., 2005a). These cellular insults are reflected in a recent analysis of the proteome in *L. monocytogenes* cells exposed to bile acids under anaerobic conditions (Payne et al., 2013). The study found significant alterations in proteins associated with DNA repair, chaperone activity and oxidative stress responses including elevated levels of excinuclease ABC proteins (UvrABC), DNA mismatch repair proteins and DnaK (Payne et al., 2013).

Similarly a recent analysis of the transcriptomic response of *L. monocytogenes* to mammalian bile has provided insights into how bile may act as a specific signal during gastrointestinal transit (Quillin et al., 2011). The study demonstrated that bile exposure regulates many virulence factors in *L. monocytogenes*. In particular the work identified a TetR-type regulator [renamed bile-regulated transcription factor A (BrtA)] that senses bile (in particular the bile acid cholic acid) and regulates expression of two multidrug resistance (MDR) efflux pumps (MdrM and MdrT) that mediate bile tolerance and liver/gall bladder colonization (Quillin et al., 2011). This finding may be particularly relevant given the broader role of MdrM/T in mediating secretion of cyclic-di-AMP, a signaling molecule that triggers STING-dependent production of interferon-beta and promotes *in vivo* survival of the pathogen (Crimmins et al., 2008; Woodward et al., 2010; Schwartz et al., 2012; Burdette and Vance, 2013).

*L. monocytogenes* strains express a bile salt hydrolase (BSH) enzyme that has the potential to detoxify individual conjugated bile acids and contributes to listerial survival in the GI tract (Dussurget et al., 2002; Begley et al., 2005b). BSH is an enzyme that is specific to commensal bacteria that inhabit the GI tract and a limited number of pathogens that infect via this route (Jones et al., 2008). The importance of the enzyme for gastrointestinal survival is evidenced by the wide distribution of this activity amongst commensals (Jones et al., 2008). BSH expression in *L. monocytogenes* is under the influence of Sigma B and is strongly enhanced during growth in the intestine and following interaction with commensal lactobacilli (Archambaud et al., 2012). Recent work suggests that BSH and Sigma B may not be essential for growth of *L. monocytogenes* in the murine gall bladder (Dowd et al., 2011) where the pathogen instead relies upon expression of pathways required for nutrient acquisition or synthesis of monomeric biomolecules (Dowd et al., 2011; Faith et al., 2012). In contrast, when the pH of bile is reduced (as occurs in the duodenum) the toxicity of bile acids is increased (Begley et al., 2005a) and the pathogen requires BSH, another bile resistance protein called bile exclusion protein (BilE) (Sleator et al., 2005) and other potentially Sigma B-regulated detoxification mechanisms (Dowd et al., 2011).

## Potential effects of the food matrix and pre-adaptation

Relatively few studies have analyzed the influence of the food matrix upon subsequent infectious potential in *L. monocytogenes*. Previous physiological studies have implied that extracellular levels of glutamate, which serves as a substrate of the GAD system (Cotter et al., 2001), or carnitine, which is taken up principally by the OpuC transporter (Sleator et al., 2001; Wemekamp-Kamphuis et al., 2002), could have the potential to influence infection of the GI tract. Furthermore, acid or salt adaptation during growth in food may influence stress-hardening of the pathogen, thereby enhancing subsequent survival in the gut (Sleator et al., 2009). Both transcriptomic and proteomic approaches are being used in order to understand the molecular mechanisms that underpin cross-adaptation to a variety of stresses (Bergholz et al., 2012; Melo et al., 2013) and the interplay between different regulatory networks (Chaturongakul et al., 2011).

A number of studies have investigated the effects of acid shock upon expression of *in vivo*-relevant genes regulated by PrfA and Sigma B (including *bsh, inlA*, and *inlB*) (Phan-Thanh and Mahouin, 1999; Abram et al., 2008; Xayarath et al., 2009; Neuhaus et al., 2013). Recent work demonstrated that acid shock significantly induced these systems and enhanced the invasive capacity for epithelial cells and the ability to infect *Caenorhabditis elegans* (an alternative host model system) (Neuhaus et al., 2013). Other work has demonstrated that oxygen restriction in the growth environment significantly enhances subsequent infectivity of *L. monocytogenes* in guinea pigs (Bo Andersen et al., 2007). Indeed hypoxia has been shown to be a potent inducer of genes required for gastrointestinal survival and invasion (including *inlA* and *inlB*) (Toledo-Arana et al., 2009).

A better understanding of how environmental conditions influence the Sigma B regulon has the potential to provide added insights into adaptation and infection by pathogen. Interesting recent work has determined that light (in the form of both blue or red light) is a potent inducer of the Sigma B regulon in *L. monocytogenes* (Ondrusch and Kreft, 2011). The signal from blue light is sensed by a blue light detector (encoded by *lmo0799*) (Ondrusch and Kreft, 2011; Tiensuu et al., 2013) and a mutation in *lmo0799* enhances the red-light effect (Ondrusch and Kreft, 2011). Exposure of *L. monocytogenes* to blue light induced *inlA* and *inlB* expression and leads to enhanced invasion of epithelial cells (Ondrusch and Kreft, 2011). However, the relevance of this finding for *in vivo* infection is not yet clear. Furthermore, a recent study indicates that oscillating day and night cycles co-ordinate differentiation of *L. monocytogenes* colonies by inducing alternating opaque and translucent rings on soft agar plates. This differentiation was mediated by Lmo0799 through induction of Sigma B, though both PrfA and ActA were also required for ring formation (Tiensuu et al., 2013). Significantly, bacteria forming opaque rings demonstrated enhanced levels of exopolysaccharide (EPS) production and elevated stress resistance indicating a significant physiological adaptation which may be significant in different phases of the infectious cycle (Tiensuu et al., 2013).

## Role of the microbiota in resistance to listeria colonization

*L. monocytogenes* is a non-fastidious and tractable target for demonstrating the barrier effect of probiotic commensals upon colonization or infection. *In vitro* cell culture studies indicate that cell invasion by *L. monocytogenes* can be inhibited by a variety of commensal bacteria (Corr et al., 2007a; Gomes et al., 2012; Nakamura et al., 2012). Experiments using germ-free mice or rats also clearly demonstrate that the gastrointestinal microbiota plays an important role as a barrier to *L. monocytogenes* infection (Czuprynski and Balish, 1981; Bambirra et al., 2007; Archambaud et al., 2012).

The mechanisms by which probiotic commensals may protect against gastrointestinal pathogens include direct antagonism, immunomodulation, enhancement of epithelial barrier function and bacterial signaling events (including quorum sensing) (Corr et al., 2009). A number of these potential mechanisms have been examined in *Listeria* oral infection models. We have shown that bacteriocin production by *Lb. salivarius* UCC118 is a dominant mechanism by which this potentially probiotic commensal mediates a level of protection against oral infection in conventional mice (Corr et al., 2007b). The work indicates that bacteriocin production *in situ* may provide a mechanistic basis by which the microbiota protects against foodborne bacteria. It also represents a potential proof-of-concept for the development of bacteriocin-producing commensal strains to protect against a number of foodborne agents (Rea et al., 2010, 2011).

The study by Archambaud and coworkers which utilized germ-free and mono-colonized E-cad^hum^ mice demonstrated the significant crosstalk that occurs between probiotic commensals and both the host and the pathogen (Archambaud et al., 2012). In particular the study revealed that probiotic commensal strains modulate the host interferon response against *L. monocytogenes* and reduce dissemination of the pathogen in the host. The study demonstrates that crosstalk between the microbiota and the host provides a mechanism for modulating defences against the pathogen *in vivo* (Archambaud et al., 2012).

## Conclusions

Increased understanding of the mechanisms by which *L. monocytogenes* adapts to the host GI tract has the potential to inform the development of novel treatment or prevention strategies. For instance a recent small molecule screening approach indicated that fluoro-phenyl-styrene-sulfonamide (FPSS) can inhibit Sigma B activity, thereby downregulating the expression of key virulence related loci (*inlA, inlB, bsh*) (Palmer et al., 2011). There is significant evidence that *Listeria*-specific bacteriophages or phage lysins can control bacterial growth in the GI tract (Coffey et al., 2010; Mai et al., 2010). Significantly, the US FDA has recently approved the use of a bacteriophage-based additive for the control of listerial growth in foods (Coffey et al., 2010). Finally, the continued investigation of potential probiotic (Corr et al., 2007b; Archambaud et al., 2012) and prebiotic (Ebersbach et al., 2010) candidates for their ability to control *L. monocytogenes* in the GI tract should inform the future development of functional foods with the potential to reduce disease incidence.

### Conflict of interest statement

The authors declare that the research was conducted in the absence of any commercial or financial relationships that could be construed as a potential conflict of interest.
